# Detection of an avian lineage influenza A(H7N2) virus in air and surface samples at a New York City feline quarantine facility

**DOI:** 10.1111/irv.12572

**Published:** 2018-06-30

**Authors:** Francoise M. Blachere, William G. Lindsley, Angela M. Weber, Donald H. Beezhold, Robert E. Thewlis, Kenneth R. Mead, John D. Noti

**Affiliations:** ^1^ Allergy and Clinical Immunology Branch Health Effects Laboratory Division National Institute for Occupational Safety and Health, Centers for Disease Control and Prevention Morgantown WV USA; ^2^ Disaster Science Responder Research Program National Institute for Occupational Safety and Health, Centers for Disease Control and Prevention Atlanta GA USA; ^3^ Engineering and Physical Hazards Branch Division of Applied Research and Technology National Institute for Occupational Safety and Health, Centers for Disease Control and Prevention Cincinnati OH USA

**Keywords:** Aerosol sampling, influenza A(H7N2), surface sampling, transmission

## Abstract

**Background:**

In December 2016, an outbreak of low pathogenicity avian influenza (LPAI) A(H7N2) occurred in cats at a New York City animal shelter and quickly spread to other shelters in New York and Pennsylvania. The A(H7N2) virus also spread to an attending veterinarian. In response, 500 cats were transferred from these shelters to a temporary quarantine facility for continued monitoring and treatment.

**Objectives:**

The objective of this study was to assess the occupational risk of A(H7N2) exposure among emergency response workers at the feline quarantine facility.

**Methods:**

Aerosol and surface samples were collected from inside and outside the isolation zones of the quarantine facility. Samples were screened for A(H7N2) by quantitative RT‐PCR and analyzed in embryonated chicken eggs for infectious virus.

**Results:**

H7N2 virus was detected by RT‐PCR in 28 of 29 aerosol samples collected in the high‐risk isolation (hot) zone with 70.9% on particles with aerodynamic diameters >4 μm, 27.7% in 1‐4 μm, and 1.4% in <1 μm. Seventeen of 22 surface samples from the high‐risk isolation zone were also H7N2 positive with an average M1 copy number of 1.3 × 10^3^. Passage of aerosol and surface samples in eggs confirmed that infectious virus was present throughout the high‐risk zones in the quarantine facility.

**Conclusions:**

By measuring particle size, distribution, and infectivity, our study suggests that the A(H7N2) virus had the potential to spread by airborne transmission and/or direct contact with viral‐laden fomites. These results warranted continued A(H7N2) surveillance and transmission‐based precautions during the treatment and care of infected cats.

## 1 INTRODUCTION

In December 2016, the Centers for Disease Control and Prevention (CDC) and the New York City Department of Health and Mental Hygiene (DOHMH) issued formal statements of an avian lineage influenza A(H7N2) virus outbreak at several animal care shelters in New York (NY) and Pennsylvania (PA).^1^, ^2^ The transmission of the virus to an attending veterinary staff member^3^ warranted the use of transmission‐based precautions including personal protective equipment (PPE) by all shelter staff and volunteers during the relocation and care of cats at a quarantine facility operated by the American Society for the Prevention of Cruelty to Animals (ASPCA).^1^, ^2^, ^4^ Genome analysis of the initial feline isolate (A/feline/New York/16‐040082‐1/2016)^5^ and subsequent virological characterization of the human isolate (A/New York/108/2016)^6^ showed it to be phylogenetically related to the North American lineage low‐pathogenic avian influenza (LPAI) A(H7N2) viruses circulating in live poultry markets during 1996‐2005. Recent analysis of the human A(H7N2) isolate showed it to transmit poorly in ferrets but, unlike previously identified isolates, displayed an increased ability to replicate in human airway cells.^7^ While it is unknown how the index cat was exposed to the A(H7N2) virus, the high rate of transmission among cats and concern about a possible zoonotic transmission mandated quarantine precautions.

In an effort to better understand transmission of the 2016 influenza A(H7N2) virus in felines and to establish the occupational exposure risk, the National Institute for Occupational Safety and Health (NIOSH), Disaster Science Responder Research (DSRR) Program initiated a pilot study at the quarantine facility. Aerosol and surface samples were collected from various locations within the quarantine facility. Gene sequencing was used to confirm the identity of the 2016 influenza A(H7N2) virus. Sample analysis was performed using quantitative PCR (qPCR) targeting either the Matrix gene (M1) or the H7‐specific hemagglutinin (HA) gene. To assess viral infectivity, aerosol and surface samples with elevated A(H7N2) M1 gene copies were subjected to serial passage in embryonated chicken eggs. Additionally, aliquots of some of these aerosol samples were first concentrated and then passaged in embryonated chicken eggs. Viral quantification was performed by qPCR and hemagglutination assay.

## 2 EXPERIMENTAL PROCEDURES

### 2.1 Quarantine facility

**Figure 1:**
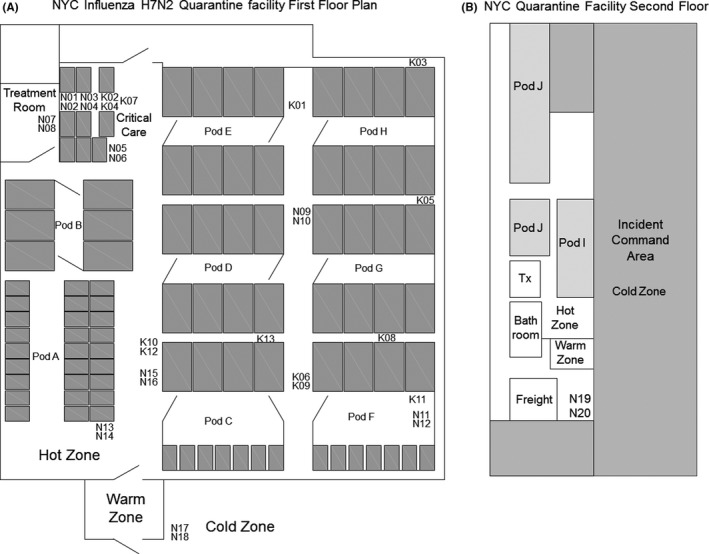
Floor plan of NYC quarantine facility. (A) The majority of cats were housed on the first floor, while (B) some were housed on the second floor. Cats were housed in cages (1‐7 cats/cage depending on the cage size). The cages were grouped into pods (6‐8 cages/pod). Cats were examined in the treatment rooms (Tx), and the sickest ones were housed individually in 15 cages, some stacked 2 high, in the critical care area. The locations of NIOSH samplers (N01‐N20) are shown. NIOSH samplers were in used in pairs with 1 sampler above the other. The locations of SKC BioSamplers (K01‐13) also are shown. SKC BioSamplers were used 1 at a time. Donning of personal protective equipment (PPE) was carried out in the cold zone. When exiting the hot zone, workers removed their PPE in the warm zone

Sampling was performed in a temporary feline quarantine facility in New York City (NYC), NY, USA, on January 5‐6, 2017. Aerosol and surface samples were collected throughout the facility's high‐risk containment area (hot zone), moderate‐risk decontamination area (warm zone), and low‐risk outside containment area (cold zone). A floor plan of the facility is shown in Figure 1A,B. At the time of sampling, the facility housed about 500 cats, about 386 of whom had tested positive for influenza A(H7N2). The quarantine facility was housed in a two‐story warehouse and had recirculating unit air heaters. The thermostat controlled unit heaters were mounted at ceiling height and cycled on/off throughout the study period. Although the recirculating unit heaters delivered no outdoor or makeup air to the space, they generated significant discharge velocities when activated, thus adding to room air turbulence and its negative influence upon aerosol settling. Outdoor air was supplied through natural ventilation that infiltrated into the facility.

The quarantine area (hot zone) where sampling was performed had a floor area of approximately 725 m^2^ (7800 ft^2^) and a ceiling height of ~3.7 m (12 feet). Cats were housed in cages, grouped into pods. Pods D, E, G, and H had eight 1.8 m × 3 m × 1.8 m (6′ × 10′ × 6′) tall cages with 3‐5 cats in each. These cages were not stacked. Pods were arranged with 4 cages on the left and 4 on the right with a central access corridor and gated entrance. Pod B had a similar arrangement but only contained 6 cages. Pod F had four 1.8 m × 3 m × 1.8 m tall cages on the left that housed 7 cats, and about 14 cages 0.9 m × 1.5 m × 0.9 m (3′ × 5′ × 3′) tall stacked 2 high that housed single cats. Cats in pod A were single‐housed in about 54 0.9 m × 1.5 m × 0.9 m tall cages in 3 rows stacked 2 high. One row was close to the wall, and the other 2 rows were back‐to‐back. Cats were examined in the treatment rooms (Tx), and the sickest ones were housed individually in 0.9 m × 1.5 m × 0.9 m tall cages stacked 2 high in the critical care area. The critical care area had 3 rows of cages. Cages in the middle and right row were stacked 2 high for a total of 6 cages in each row with an access passageway between the right and middle row. The left row was only 1 cage high for a total of 3 cages; access was from the walkway. The overall total was 15 cages in the critical care area, although not all had cats in them. Cages with cats were marked with clipboard containing paperwork hanging from the door of each cage. The locations of NIOSH samplers (N01‐N20) are shown (Figure 1A,B). NIOSH samplers were used in pairs with 1 sampler above the other. The locations of SKC BioSamplers (K01‐13) also are shown (Figure 1A). SKC BioSamplers were used 1 at a time.

During sampling, the interior temperature was 18°C and the relative humidity was 25%. All workers entered and exited the hot zone through the warm zone. Before entering the hot zone, workers donned disposable coveralls, N95 respirators, hair covers, shoe covers, eye protection, and 2 pairs of gloves. After leaving the hot zone, workers removed their personal protective equipment in the warm zone.

### 2.2 Aerosol sample collection

Aerosol samples were collected using NIOSH BC 251 two‐stage cyclone samplers (denoted as NXX) and SKC BioSamplers (denoted as KXX). Sampling locations are shown in Figure 1A,B.

Twenty aerosol samples (N01‐08 on day 1, N09‐20 on day 2) were collected using the NIOSH BC 251 sampler^8^ at a 3.5 L/min flow rate, except for 2 samples (N02 and N03), which were inadvertently collected at 3.8 and 3.9 L/min. The NIOSH sampler separates the particles into 3 size fractions (≥4 μm, 1 to 4 μm, and ≤1 μm) and conforms to the American Conference of Governmental Industrial Hygienists/International Organization for Standardization criteria for respirable particle sampling.^9^ The samplers were mounted on tripods in pairs, with the odd‐numbered sampler above the even‐numbered 1. The sampler inlet heights ranged from 22 to 211 cm above the floor. The sampling lasted for 4‐5 hours, except for 1 sample (N15) for which the pump shut down after 59 minutes. Sixteen of the samples (N01‐N16) were collected inside the first floor hot zone, while two (N17 & N18) were collected just outside the entrance to the warm zone. Two additional samples (N19 and N20) were collected just outside the entrance to the second floor warm zone. After aerosol sampling was completed, aerosol samples were suspended on‐site in Lysis/Binding Solution Concentrate (ThermoFisher Scientific, Pittsburgh, PA, USA). One mL of lysis concentrate was added to the >4 μm and <1 μm aerosol fractions stages, while 0.4 mL was added to the 1‐4 μm aerosol fraction. All samples were stored on ice for 1‐2 days before transport to the laboratory for analysis.

Thirteen aerosol samples (K01‐03 on day 1, K04‐13 on day 2) were collected using SKC BioSamplers (SKC, Eighty Four, PA, USA) at a nominal flow rate of 12.5 L/min. The particle collection efficiency for the SKC BioSampler (i.e., the percentage of particles of a given size that are collected by the sampler) is reported to be ~10% for particles with aerodynamic diameters of 0.1 μm; 50% for 0.3‐μm particles; 96% for 1‐μm particles; 100% for 2‐μm particles; and 50% for 8‐μm particles.^10^, ^11^, ^12^ Particles larger than 10‐15 μm are expected to be removed by the sampler elbow and not collected. The biosamplers contained 20 mL of viral transport media (VTM) consisting of Hank's balanced salt solution (HBSS; ThermoFisher Scientific) supplemented with 0.1% bovine serum albumin (BSA; Sigma‐Aldrich, St. Louis, MO, USA), 100 units/mL penicillin G and 100 units/mL streptomycin (ThermoFisher Scientific). Sampling was halted every 15 minutes during collection, and sterile distilled water was added as needed to the collection vessel to replace water lost due to evaporation. The biosamplers were mounted individually on tripods during collection with the inlets 46‐52 cm above the floor, and all samples were collected for 1 hour at locations inside the hot zone. After aerosol collection was completed, samples were placed on ice and transported to the laboratory after 1‐2 days.

### 2.3 Surface sample collection

Thirty‐one surface samples were collected on day 1 of sampling using sterile nylon‐flocked swabs (Copan Diagnostics, Corona, CA, USA) moistened with VTM. Surface samples are denoted as SXX. Samples were collected from areas with direct animal contact such as cages/crates, floors, and water bowls, and from common worker high‐contact porous and non‐porous surfaces such as door knobs, table tops, and elevator buttons. When possible, a 10 cm × 10 cm cardboard template was placed on flat surfaces and surface sampling was carried out by swiping a swab completely across the template in 3 directions (left to right, perpendicular, and diagonal). In some cases (sampling doorknobs, elevator buttons, water bowls), an area ~50‐100 cm^2^ was sampled. The sampling method had not been robustly evaluated or validated due to time constraints on obtaining samples. After collection, swabs were placed in 0.5 mL VTM and kept on ice until transport to the laboratory after 1‐2 days.

### 2.4 Propagation in chicken eggs

Due to the inability of A/feline/NY/2016 to efficiently replicate in cell culture, viability was assessed using fertile White Leghorn chicken eggs (serum pathogen free) (Charles River, MA). Aerosol and surface swab samples that were found to be A(H7N2) qPCR positive with elevated M1 copy numbers were inoculated in duplicate into 10‐day‐old embryonated chicken eggs (incubated at 37°C, 45% humidity, MX‐20, R‐COM, Wichita, KS). With SKC aerosol samples, 0.2 mL of undiluted sample was inoculated into the allantoic cavity of each egg, whereas with surface samples, a 0.125 mL inoculum volume (undiluted) was used. For sequential passage in embryonated chicken eggs, 0.2 mL of sample allantoic fluid was used as the inoculum source. Following inoculation, eggs were incubated for 48 hours at 35°C, 50% humidity. Prior to harvesting the allantoic fluid, eggs were chilled overnight at 4°C to terminate embryos and restrict flow of blood into the allantoic cavity. Collection of allantoic fluid was performed according to Current Protocols in Microbiology 15G.1.6. The final volume of allantoic fluid collected for each inoculated egg sample was ~ 6 mls.

### 2.5 InnovaPrep concentration

To establish the presence of infectious virus on airborne particles, selected SKC BioSampler samples were concentrated using the InnovaPrep Concentrating (IPC) Pipette. SKC aerosol sample K03 (15 mL) was drawn through a single disposable ultrafiltration concentrating pipette tip (InnovaPrep, LLC., Drexel, MO, USA) and extracted to a final volume of 0.7 mL, using the manufacturer supplied DMEM/N2O elution fluid. To increase the likelihood of detecting infectious virus, samples K01, K02, and K08 were first pooled together (Final volume ~20 mL) and similarly run through an ultrafiltration concentrating pipette tip. The final extracted sample volume was 0.6 mL. A 0.2 mL volume of each IPC sample was injected into individual 10‐day‐old embryonated chicken eggs. Quantitative PCR detection of the M1 gene was used to quantify viral loads from the resultant allantoic fluid.

### 2.6 Viral RNA isolation

#### 2.6.1 NIOSH two‐stage cyclone samples

Viral RNA was extracted from NIOSH two‐stage cyclone samplers using the MagMAX‐96 Viral RNA Isolation Kit (ThermoFisher Scientific) as previously described.^8^ Upon thawing and resuspension by vortexing 1 minute, samples were supplemented with a 1:1 volume of 2‐propanol (Sigma) followed by the addition of 1 μL carrier RNA and 20 μL of prepared bead mix. Viral RNA was washed and processed according to the manufacturer's protocol. Eluted viral RNA in total (26.4 μL) was immediately transcribed to cDNA.

#### 2.6.2 SKC samples

For samples collected with the SKC BioSampler, a 2 mL volume (10% of collected sample) was used for viral RNA isolation. To account for the larger sample volume, a modified MagMAX‐96 Viral RNA isolation protocol was utilized. Briefly, samples were extracted using a 1:1 volume of 2‐propanol (Sigma) followed by the addition of 2 μL carrier RNA and 30 μL of prepared magnetic bead mix. Based on the magnetic properties of the nucleic acid‐binding beads, sample volumes were reduced while viral RNA was captured. The bead‐bound viral RNA was then washed and processed according to the manufacturer's protocol. The entire volume of eluted viral RNA (26.4 μL) was immediately transcribed to cDNA.

#### 2.6.3 Surface samples

For surface samples, a 0.25 mL volume (50% of collected sample) was used for viral RNA isolation. Following the 1:1 addition of 2‐propanol, samples were spiked with 1 μL carrier RNA, 20 μL of prepared bead mix and processed according to the MagMAX‐96 Viral RNA Isolation Kit. Isolated viral RNA was eluted with 26.4 μL elution buffer and immediately transcribed to cDNA.

#### 2.6.4 Allantoic fluids

Viral RNA was extracted from 1 mL of allantoic fluid derived from surface swab, SKC, and concentrated SKC samples that were passaged in embryonated chicken eggs. The MagMAX‐96 Viral RNA isolation Kit protocol was modified comparably to the methods used in isolating viral RNA from SKC aerosol samples. The isolated viral RNA was immediately transcribed to cDNA.

### 2.7 cDNA transcription

Isolated viral RNA was transcribed to complimentary DNA using the High‐Capacity cDNA Reverse Transcription Kit (ThermoFisher Scientific) as described by the manufacturer's protocol. Final cDNA volume on all aerosol, surface swab, and allantoic samples was 40 μL.

### 2.8 pDNA standards

To quantify Matrix (M1) gene copies during the qPCR analysis of samples, a plasmid DNA standard was used as described previously.^13^ For H7 HA‐specific quantification, the complete DNA sequence of the hemagglutinin gene for A/feline/NY/2016 was synthesized and cloned into pUC57‐Kan by Genewiz (Genewiz Ltd., South Plainfield, NJ, USA). The resultant HA‐pDNA, designated pJAB#1, was linearized by the restriction endonuclease enzyme *Xho*I, purified using the QIAquick PCR Purification Kit according to manual procedures (Qiagen, Hilden, Germany) and quantified by spectrometry (NanoDrop 2000, Thermo Scientific). To quantify influenza HA copies, a standard curve was generated by performing 10‐fold dilutions on the linearized, purified HA‐pDNA.

### 2.9 Quantitative qPCR

Quantitative PCR of the Matrix M1 gene was performed as previously described.^13^ For qPCR detection of the influenza A/feline/NY/H7N2/2016 variant, Primer Express Software v2.0 (ThermoFisher Scientific) was used to design primers and a probe for H7‐HA‐specific detection. Synthesis of the primers and probe was performed by Applied Biosystems (ThermoFisher Scientific). DNA sequences for HA‐qPCR detection are as follows: H7HA +1244 forward (5′ ATTGGACACGAGACGCAATG 3′), H7HA −1342 reverse (5′ TTCTGA GTCCGCAAGATCTATTG 3′) and H7HA +1281 Probe (5′ 6FAM‐TAATGCTGAGCTGTTGGTGGCA‐MGBNFQ). Primer and probe concentrations were 0.9 μm and 0.25 μm, respectively. All reactions were performed in duplicate and analyzed using the Applied Biosystems 7500 Fast Real‐Time PCR System under the following cycling conditions: 95°C for 20 seconds followed by 45 cycles at 95°C for 3 seconds and 60°C for 30 seconds. A no template control was run in parallel with all qPCR assays.

### 2.10 DNA sequence analysis

For the verification of A/feline/NY/2016, selected qPCR‐positive aerosol and surface swab samples were purified according to protocol using the QIAquick PCR Purification kit (Qiagen) and submitted to Genewiz for Sanger DNA sequencing services. Sequence analysis was performed on both the 5′ and 3′ end of submitted DNA using the above mentioned M1 and A(H7N2) HA oligonucleotides.

### 2.11 Hemagglutination assay and stock viruses

As a measure of viral replication, hemagglutination assays were performed on the collected allantoic fluid as described by Current Protocols in Microbiology 15G.1.8. Chicken red blood cells (RBCs) were obtained from Lampire Biological Laboratories (Everett, PA, USA) and prepared to a final concentration of 0.5%. Briefly, 50 μL of sample allantoic fluid was serially diluted in twofold increments using 1X PBS (ThermoFisher Scientific) followed by the addition of 50 μL 0.5% chicken RBCs. Plates were then incubated for at least 30 minutes at room temperature before agglutination was assessed. Positive HA controls A/NY/108/2016 (H7N2) and A/feline/NY/2016 (H7N2) were kindly provided by the CDC, Atlanta, and University of Wisconsin, respectively.

## 3 RESULTS

### 3.1 Aerosol sampling in the quarantine facility

The cat quarantine facility was a two‐floor warehouse with the majority of cats housed in the hot zone on the first floor (Figure 1A) and the remainder in the smaller second floor hot zone (Figure 1B) that was adjacent to the office/meeting space for the ASPCA workers. To assess the risk of occupational exposure to influenza‐containing aerosols at the cat quarantine facility, aerosol samples were collected over 2 days using NIOSH aerosol samplers, which separate and collect aerosols into 3 size fractions (>4 μm, 1‐4 μm, and <1 μm aerodynamic diameters), and biosamplers, which collect airborne particles into viral transport media.

**Figure 2:**
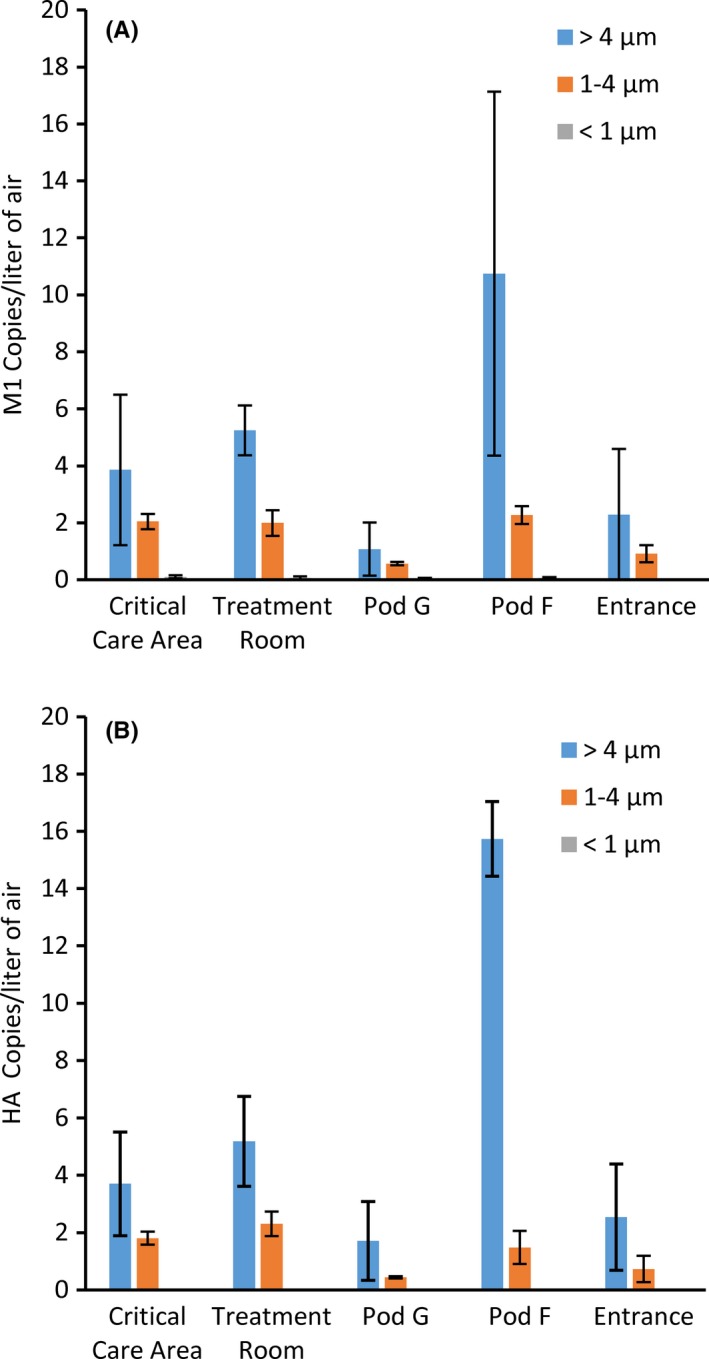
Aerodynamic size distribution of particles containing H7N2 in the hot zone. The size distribution of (A) M1 and (B) HA gene copies in aerosol samples collected with the NIOSH 2‐stage sampler. Sixteen NIOSH samplers were positioned throughout the hot zone of the first floor quarantine facility. The samplers were mounted in pairs on a tripod and each sampler collected air for 260‐307 min. Critical care area (samplers N01‐06), treatment room (N07, N08), Pod G (N09, N10), Pod F (N11, N12), entrance (N13‐16). The average M1 and HA gene copies/L of air (±standard deviation) collected in each aerosol fraction are shown

**Figure 3:**
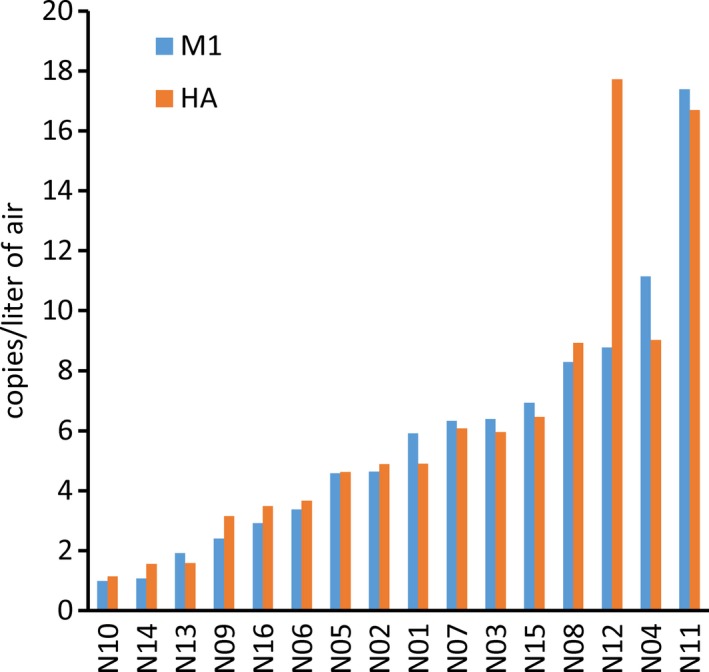
Total H7N2 virus collected by NIOSH two‐stage samplers in the hot zone of the quarantine facility. The average M1 and HA gene copies/L of air collected from all 3 aerosol fractions (>4 μm, 1‐4 μm, and <1 μm) by the 16 samplers (N01‐16) positioned in the first floor of the quarantine facility are shown

Sixteen NIOSH aerosol samplers were positioned throughout the first floor hot zone, 2 samplers placed in the first floor cold zone, and two in the second floor cold zone. Each NIOSH sampler collected airborne particles for 260‐307 minutes. The arithmetic average M1 and HA copies collected over 260‐307 minutes within specific regions of the quarantine facility are shown in Figure 2A,B. Results for individual samplers are shown in Figure 3, supplemental Tables S1 and S2. For samples collected in the critical care area, the average M1 copies/L of air detected in the >4 μm, 1‐4 μm, and <1 μm size fractions were 3.86, 2.04, and 0.10, respectively. Similarly, 3.70, 1.80, and 0.00 HA copies/L of air were detected from the equivalent size fractions. Comparable M1 (5.25, 1.99, and 0.06) and HA (5.19, 2.31, and 0.00) gene copies/L were detectable from aerosol fractions collected in the treatment room. In aerosol samples collected in Pod F, the greatest M1 (10.74) and HA (15.73) gene copies/L were detected in the >4 μm fraction, while 2.27 (M1) and 1.48 (HA) gene copies/L were detectable in the 1‐4 μm size fraction. In the <1 μm fraction, 0. copies/L were detected in Pod F, while the same fraction was HA‐qPCR undetectable. In front of Pod G, 1.08 and 0.57 M1 and 1.71 and 0.43 HA gene copies/L were detected from aerosol particles collected in the >4 μm and 1‐4 μm fraction, respectively, while only 0.05 M1 gene copies/L were detectable from the <1 μm fraction. Lastly, aerosol samples collected in the hot zone adjacent to the entrance from the warm zone had an average of 2.29, 0.91, and 0.01 M1 copies/L in the 3 size fractions, while 2.54 and 0.73 HA copies/L were detected in the >4 μm and 1‐4 μm fraction and HA not detected in the <1 μm aerosol fraction. A low level of M1 (but not HA) was detected in the >4 μm fraction from 1 sample (N20) in the second floor cold zone, while the other 3 samples from the cold zones were negative. DNA sequence analysis showed that the M1 and HA PCR products of virus collected in the >4 μm and 1‐4 μm aerosol fractions were 100% homologous to the M1 and HA nucleotide sequences of the cat (A/feline/New York/16‐040082‐1/2016) and human (A/New York/108/2016) isolates from the NYC A(H7N2) outbreak (data not shown).

To maintain virus infectivity, biosamplers, which directly deposit bioaerosols in a liquid reservoir, were used to collect aerosol samples throughout quarantine hot zones. Thirteen biosamplers were positioned throughout the first floor hot zone over 2 days and collected aerosols for 60 minutes. As the M1 and HA‐qPCR analyses of the samples collected using the NIOSH aerosol samplers gave comparable results, M1 qPCR was exclusively performed on samples collected using the biosamplers. The highest amount of virus was collected in samples K01, K06, and K08 (5.97 arithmetic mean copies, SD = 0.47 M1 copies/L air). The lowest amount was collected in samples K02, K07, K09, and K12 (1.28 mean copies, SD = 0.46 M1 copies/L of air). Results from individual samplers are shown in supplemental Table S3.

Because A/feline/NY/2016 (H7N2) failed to infect/replicate in either Madin Darby canine kidney (MDCK) or Crandell Reese feline (CRFK) cells, quantification by viral plaque assay was not possible (results not shown). To assess whether the collected virus was infectious, aliquots from samples K01, K02, K03, K05, K12, and K13 were inoculated into 10‐day‐old embryonated chicken eggs and A(H7N2) M1 gene copies were measured after 48 hours. Infectious virus was found in all samples, and overall M1 gene copies increased fivefold to 223‐fold (Table 1). Aliquots of the allantoic fluid from the first passage of virus derived from samples K01, K02, and K03 were passaged again in eggs and M1 copies increased 11‐ to 112‐fold (Table 1). Increasing the A(H7N2) egg inoculum from pooled samples K01, K02, and K08 in an InnovaPrep Concentrating Pipette by approximately 33‐fold led to a 47‐fold increase in M1 copies. Likewise, concentrating sample K03 approximately 22‐fold resulted in a dramatic 2 × 10^7−8^‐fold increase in M1 copies and enabled hemagglutination detection with a final HA titer of 512 HA units (HAU)/50 μL allantoic fluid (Table 2).

**Table 1:** M1 gene copies in egg‐propagated SKC samples—1st and 2nd passages

Sample ID	Location	1st Passage			2nd Passage			
		M1 Copies in 0.2 mL inoculum	M1 copies in 6 mL allantoic fluid	Fold increase in M1 copies	M1 Copies in 0.2 mL inoculum	M1 copies in 6 mL allantoic fluid		Fold increase in M1 copies
K01	Outside of Pod E	49.9	259	5	8.60	Egg A Egg B	112 904	13 105
K02	Critical care room	12.6	619	49	20.6	Egg A Egg B	431 228	21 11
K03	Outside of Pod H	34.0	499	15	16.6	Egg A	1860	112
K05	Outside of Pod G	26.7	1150	43				
K12	Outside of Pod C	13.8	1300	94				
K13	Between Pods C and D	26.3	5870	223				

**Table 2:** M1 gene copies in egg‐propagated InnovaPrep Concentrated (IPC) K samples

Sample ID	Location	M1 copies in 0.2 mL inoculum	M1 copies in 6 mL allantoic fluid	Fold increase in M1 copies
IPC K01	Between Pods E and H	1.22 × 10^3^	5.69 × 10^4^	47
IPC K02	Critical care room			
IPC K08^a^	Between Pods F and G			
IPC K03‐A^b^	Outside of Pod H	7.3 × 10^2^	1.47 × 10^11^	2.0 × 10^8^ c
IPC K03‐B^b^	Outside of Pod H	7.3 × 10^2^	1.48 × 10^10^	2.0 × 10^7^ c

a Pooled K01, K02, and K08 samples were concentrated to a final volume of 0.6 mL.

b Sample K03 was concentrated to a final volume of 0.7 mL. Uppercase A, B denotes inoculated egg replicate.

c Hemagglutination analysis yielded 512 HAU/50 μL of allantoic fluid.

### 3.2 Surface sampling in the quarantine facility

PCR analysis of surface samples from quarantine hot zones showed an average M1 copy number of 885/~100 cm^2^, comparable to M1 copies found in the >4‐μm and 1‐ to 4‐μm particle fractions after approximately 4‐6 hours of air sampling with the NIOSH samplers. Surface samples S64, S73, S79, and S89 (obtained from Pod J, Pod G, and the floor near the entrance from the hot zone) had the most M1 copies, ranging from 1336 to 9516 (results for individual surface swabs are shown in supplemental Table S4). Surface samples taken from cold zone areas showed no detectable M1 copies. To determine whether infectious virus was also present in surface swab samples from the hot zones, aliquots of swab samples S64, S73, S75, S79, and S89 were passaged in 10‐day‐old embryonated chicken eggs and A(H7N2) titers were measured after 48 hours. Infectious virus was found in all 5 swab samples, and overall titers increased 26‐651‐fold (Table 3). Aliquots of the allantoic fluid from the first passage of virus‐derived swab samples S75 and S89 were passaged again in eggs and titers increased 42‐46‐fold (Table 3).

**Table 3:** M1 gene copies in egg‐propagated surface samples—1st and 2nd passages

Sample ID	Location	1st Passage				2nd Passage			
		M1 copies in 0.125 mL inoculum	M1 copies in 6 mL allantoic fluid		Fold increase in M1 copies	M1 copies in 0.125 mL inoculum	M1 copies in 6 mL allantoic fluid		Fold increase in M1 copies
S64	Hot zone, Pod J, paper inside cage	470	Egg A Egg B	1.36 × 10^5^ 8.02 × 10^4^	289 171				
S73	Hot zone, Pod G, inside floor by cat	335	Egg A	3.77 × 10^4^	113				
S75	Hot zone, Pod F, cardboard box with cat inside	172	Egg A Egg B	1.12 × 10^5^ 6.61 × 10^4^	651 384	2330 1380	Egg A Egg B	1.05 × 10^5^ 5.82 × 10^4^	45 42
S79	Hot zone, Pod G, surface of paper inside	2380	Egg A Egg B	6.09 × 10^4^ 6.92 × 10^4^	26 29				
S89	Hot zone, concrete floor near entrance	1030	Egg A	6.89 × 10^4^	67	1440	Egg A	6.63 × 10^4^	46

## 4 DISCUSSION AND CONCLUSION

The potential for the A(H7N2) virus crossing the species barrier aptly raises concern. Though the LPAI A(H7N2) subtype is primarily found in wild and domestic birds, the 2016 feline influenza A(H7N2) outbreak is the first time A(H7N2) has been detected and transmitted among cats. Like several animal species, cats express both α2‐3 and α2‐6 sialic acid‐linked receptors on cells,^14^ and may contribute to the emergence of a novel avian A(H7N2) virus capable of binding to the predominant α2‐6 linked sialic receptors in the human respiratory tract.^15^ Moreover, the ability of H7 viruses to acquire polybasic amino acids at the HA cleavage site creating a more pathogenic virus^16^ coupled with the transmission of this recent feline A(H7N2) virus to a veterinarian in direct contact with the NYC infected cats warranted strict quarantine precautions in an effort to reduce disease transmission among cats and humans.

The average concentration of virus as determined by the number of M1 copies was 5.81 copies/L of air within the hot zone of the main floor. This concentration of A(H7N2) in the quarantine facility is comparable to H1N1 levels during influenza season found by Lindsley et al^17^ in an urgent care medical clinic (12 virus/L of air), Yang et al^18^ in a healthcare facility (16 virus/L of air), and Tseng et al^19^ in a pediatric emergency room (0.17‐5 virus/L of air). Together M1 and HA‐qPCR analysis showed that 75% of viral RNA was present on particles ≥4 μm in aerodynamic diameter. Particles from 4 to 10 μm can remain airborne for an extended time, while larger particles settle more quickly and tend to deposit near the source. Approximately 25% of the virus collected by the NIOSH samplers was on particles smaller than 4 μm, which are respirable; that is, they can remain airborne for an extended time and can be drawn deeply into the lungs when inhaled.^9^, ^20^ Thus, our results support both airborne and fomite‐mediated A(H7N2) transmission as potential transmission routes among cats. Because the deposition site of any respiratory pathogen within the airway ultimately affects disease kinetics and pathogenesis, transmission‐based precautions were pivotal to protecting quarantine staff and volunteers.

The reported survival of influenza virus on surfaces varies with the type of surface and how long the virus resides on the surface.^21^, ^22^ Housing and attending to this large population of cats made it likely that a variety of surfaces within the facility would be contaminated. On average, 885 M1 copies/~100 cm^2^ surface were detected by qPCR from swab samples taken from quarantine hot zones whereas A(H7N2) virus was undetectable in 100% of the cold zone swab samples. Samples collected from the paper used to line the cage floors, a cardboard box housing an infected cat, and the concrete floor within the hot zone were serially‐passaged in embryonated chicken eggs and M1 gene levels were measured using qPCR. M1 gene levels increased 26 to 651‐fold after a single passage in embryonated eggs, showing that the virus remained infectious on all the different surfaces.

Concern for occupational transmission of this virus to workers at the quarantine facility prompted this 2‐day study. By measuring particle size, distribution, and infectivity, our study suggests that A(H7N2) transmission had the potential to occur through aerosol transmission of small viral‐laden respiratory droplets and/or contact with viral‐laden fomites. Belser et al^7^ investigated the relative risk of transmission among workers using a ferret model of transmission and showed that ferrets intranasally inoculated with the A/NY/108/2016 virus became productively infected but did not transmit the virus to ferrets placed in an adjacent cage. However, naïve ferrets placed in the same cage with infected ferrets showed low‐titer virus in the nasal washes but did not seroconvert. The authors suggested that low‐level spread of virus between cohoused ferrets occurs in the absence of productive infection. Hatta et al^23^ assessed transmission of the A/feline/NY/2016 in ferrets and cats. In contrast to Belser et al^7^ 1 of 3 pairs of ferrets placed in direct contact showed productive infection and seroconversion but no respiratory droplet transmission to ferrets placed in adjacent cages. They suggested that amino acid differences in the PA, HA, and NA proteins between the human and cat isolates or the small group size may account for this difference.

Belser et al^7^ also showed that direct aerosol inhalation of a high dose but not a low dose of virus led to productive infection but, as with nasal inoculation, transmission to cohoused contact ferrets did not result in their seroconversion. In an earlier study, Belser et al^24^ additionally showed that infection via the ocular route was possible in ferrets although they did not assess transmission. Overall, regardless of inoculation route, the virus was of low virulence and poorly transmissible among ferrets. Belser et al^7^ also revealed that A/NY/108/2016 did not demonstrate a tropism for ferret tracheal cells but this strain exhibited increased replication efficiency in human bronchial epithelial cells compared to other A(H7N2) isolates, which poses a concern for potential human transmission. Given that human transmission occurred in 1 individual at the NYC quarantine facility, further adaptation potentially could lead to increased human transmission.

Transmission of influenza is complex and dependent upon a myriad of events. While both domestic and wild cats have been shown to be infected by influenza A virus subtypes H1N1, H3N2 as well as the HPAI H5N1 subtype, the 2016 feline A(H7N2) subtype marks the first of its kind. Despite low pathogenicity in ferrets, the A(H7N2) viral outbreak quickly spread to most of the cats in the quarantine facility. Hatta et al^23^ showed that A/feline/NY/2016 can, although inefficiently, transmit among cats via contact and respiratory droplet transmission. The high rate of transmission of A/feline/NY//2016 may, in part, reflect the large number of animals in the facility and the lack of central ventilation and/or dedicated exhaust systems to mitigate the high potential for continued suspension and reaerosolization of the virus. As cats typically exhibit a low cough volume of approximately 84 mL,^25^ recovery of virus from predominantly larger aerosol particles may have been a result of virus initially deposited on fomites (such as bedding, litter, and the paper lining the cages) that subsequently became aerosolized during the daily cage cleanup or by the activities of the cats themselves. These reaerosolized particles might have been inhaled by the cats or transferred from the PPE of shelter workers as they handled multiple cats throughout the day. While person‐to‐person transmission was not observed, LPAI viruses like the 2016 feline A(H7N2) strain are capable of eliciting mild illness in humans and may pose a pandemic threat. Prompt risk assessment of any pathogenic microorganism is key to preventing occupational exposure, controlling disease transmission, and maintaining public health.
