# Supplementary material for: Detection of an avian lineage influenza A(H7N2) virus in air and surface samples at a New York City feline quarantine facility
Source: Influenza Other Respir Viruses. 2018 Jun 30;12(5):613–22. doi: 10.1111/irv.12572 (PMC6086858; doi:10.1111/irv.12572)
Supplement: Supplementary file 1 [file IRV-12-613-s001.docx]

# Detection of an Avian Lineage Influenza A(H7N2) Virus in Air and Surface Samples at a New York City Feline Quarantine Facility

# Francoise M. Blachere, William G. Lindsley, Angela M. Weber, Donald H. Beezhold, Robert E. Thewlis, Kenneth R. Mead, and John D. Noti

# Supplemental information

Table S1. Location of NIOSH Samplers and distribution of M1 gene copies per liter of air.

| **NIOSH**  **sampler ID** | **Sampler location** | **Inlet height**  **above floor (cm)** | **Liters of air**  **collected** |  | **M1 copies per L of air** | | | |
| --- | --- | --- | --- | --- | --- | --- | --- | --- |
|  |  |  |  |  | **> 4 µm** | **1-4 µm** | **< 1 µm** | **Total** |
| N01 | Hot Zone, Critical Care Area | 95 | 942 |  | 4.14 | 1.61 | 0.16 | 5.91 |
| N02 | Hot Zone, Critical Care Area | 22 | 942 |  | 2.45 | 2.07 | 0.11 | 4.63 |
| N03 | Hot Zone, Critical Care Area | 92 | 959 |  | 4.06 | 2.25 | 0.07 | 6.39 |
| N04 | Hot Zone, Critical Care Area | 22 | 952 |  | 8.73 | 2.36 | 0.05 | 11.14 |
| N05 | Hot Zone, Critical Care Area | 97 | 977 |  | 2.63 | 1.89 | 0.06 | 4.58 |
| N06 | Hot Zone, Critical Care Area | 24 | 956 |  | 1.12 | 2.06 | 0.18 | 3.36 |
| N07 | Hot Zone, Treatment Room | 64 | 910 |  | 4.63 | 1.68 | 0.02 | 6.33 |
| N08 | Hot Zone, Treatment Room | 26 | 910 |  | 5.87 | 2.31 | 0.11 | 8.29 |
| N09 | Hot Zone, Front of Pod G | 150 | 1085 |  | 1.73 | 0.61 | 0.06 | 2.4 |
| N10 | Hot Zone, Front of Pod G | 58 | 1085 |  | 0.42 | 0.53 | 0.04 | 1.0 |
| N11 | Hot Zone, Back of Pod F | 150 | 1075 |  | 15.26 | 2.05 | 0.09 | 17.4 |
| N12 | Hot Zone, Back of Pod F | 58 | 1075 |  | 6.22 | 2.49 | 0.04 | 8.76 |
| N13 | Hot Zone, near entrance from warm zone | 145 | 1043 |  | 1.0 | 0.90 | 0.02 | 1.91 |
| N14 | Hot Zone, near entrance from warm zone | 56 | 1043 |  | 0.45 | 0.63 | 0.00 | 1.08 |
| N15 | Hot Zone, near entrance from warm zone | 211 | 207 |  | 5.56 | 1.33 | 0.00 | 6.92 |
| N16 | Hot Zone, near entrance from warm zone | 173 | 980 |  | 2.12 | 0.80 | 0.00 | 2.93 |
| N17 | Cold Zone, near entrance from warm zone | 157 | 1026 |  | 0.00 | 0.00 | 0.00 | 0.00 |
| N18 | Cold Zone, near entrance from warm zone | 132 | 1026 |  | 0.00 | 0.00 | 0.00 | 0.00 |
| N19 | Cold Zone, near entrance from warm zone | 152 | 1036 |  | 0.00 | 0.00 | 0.00 | 0.00 |
| N20 | Cold Zone, near entrance from warm zone | 57 | 1036 |  | 0.12 | 0.00 | 0.00 | 0.12 |

Table S2. Location of NIOSH samplers and distribution of HA gene copies per liter of air.

| **NIOSH**  **sampler ID** | **Sampler location** | **Inlet height**  **above floor (cm)** | **Liters of air**  **collected** |  | **HA copies per L of air** | | | |
| --- | --- | --- | --- | --- | --- | --- | --- | --- |
|  |  |  |  |  | **> 4 µm** | **1-4 µm** | **< 1 µm** | **Total** |
| N01 | Hot Zone, Critical Care Area | 95 | 942 |  | 3.37 | 1.53 | 0.01 | 4.91 |
| N02 | Hot Zone, Critical Care Area | 22 | 942 |  | 2.83 | 2.05 | 0.00 | 4.88 |
| N03 | Hot Zone, Critical Care Area | 92 | 959 |  | 4.14 | 1.81 | 0.00 | 5.96 |
| N04 | Hot Zone, Critical Care Area | 22 | 952 |  | 7.11 | 1.90 | 0.00 | 9.01 |
| N05 | Hot Zone, Critical Care Area | 97 | 977 |  | 2.63 | 1.98 | 0.01 | 4.62 |
| N06 | Hot Zone, Critical Care Area | 24 | 956 |  | 2.13 | 1.52 | 0.00 | 3.66 |
| N07 | Hot Zone, Treatment Room | 64 | 910 |  | 4.07 | 2.01 | 0.00 | 6.08 |
| N08 | Hot Zone, Treatment Room | 26 | 910 |  | 6.30 | 2.61 | 0.01 | 8.92 |
| N09 | Hot Zone, Front of Pod G | 150 | 1085 |  | 2.69 | 0.46 | 0.00 | 3.15 |
| N10 | Hot Zone, Front of Pod G | 58 | 1085 |  | 0.74 | 0.41 | 0.00 | 1.15 |
| N11 | Hot Zone, Back of Pod F | 150 | 1075 |  | 14.81 | 1.89 | 0.00 | 16.70 |
| N12 | Hot Zone, Back of Pod F | 58 | 1075 |  | 16.65 | 1.07 | 0.00 | 17.72 |
| N13 | Hot Zone, near entrance from warm zone | 145 | 1043 |  | 1.31 | 0.28 | 0.00 | 1.59 |
| N14 | Hot Zone, near entrance from warm zone | 56 | 1043 |  | 1.02 | 0.53 | 0.00 | 1.55 |
| N15 | Hot Zone, near entrance from warm zone | 211 | 207 |  | 5.08 | 1.36 | 0.00 | 6.45 |
| N16 | Hot Zone, near entrance from warm zone | 173 | 980 |  | 2.75 | 0.73 | 0.00 | 3.48 |
| N17 | Cold Zone, near entrance from warm zone | 157 | 1026 |  | 0.00 | 0.00 | 0.00 | 0.00 |
| N18 | Cold Zone, near entrance from warm zone | 132 | 1026 |  | 0.00 | 0.00 | 0.00 | 0.00 |
| N19 | Cold Zone, near entrance from warm zone | 152 | 1036 |  | 0.00 | 0.00 | 0.00 | 0.00 |
| N20 | Cold Zone, near entrance from warm zone | 57 | 1036 |  | 0.00 | 0.00 | 0.00 | 0.00 |

Table S3. Location of SKC BioSamplers and M1 gene copies per liter of air. All samples were collected in the hot zone

| **Biosampler ID** | **Sampler Location** | **Inlet height**  **above floor (cm)** | **Liters of air**  **collected** | **M1 Copies**  **per L of air** |
| --- | --- | --- | --- | --- |
| K01 | Outside of Pod E | 46 | 768 | 6.5 |
| K02 | Critical Care Room | 52 | 828 | 1.5 |
| K03 | Outside of Pod H | 50 | 834 | 4.1 |
| K04 | Critical Care Room | 46 | 768 | 2.0 |
| K05 | Outside of Pod G | 48 | 828 | 3.2 |
| K06 | Outside of Pod F | 46 | 774 | 5.8 |
| K07 | Critical Care Room | 46 | 768 | 0.6 |
| K08 | Between Pods F and G | 48 | 762 | 5.6 |
| K09 | Outside of Pod F | 46 | 816 | 1.4 |
| K10 | Outside of Pod C | 46 | 762 | 0.0 |
| K11 | Outside of Pod F | 46 | 750 | 2.3 |
| K12 | Outside of Pod C | 46 | 858 | 1.6 |
| K13 | Between Pods C and D | 47 | 750 | 3.5 |

Table S4. Sample location and M1 gene copies per surface sample.

| **Surface**  **sample ID** | **Containment zone** | **Collection location** | **Area Sampled (cm^2^)** | **Total M1 copies**  **per surface sample** |
| --- | --- | --- | --- | --- |
| S01 | Hot | Critical care-plastic crate housing cats in cage 10 | ~50-100 | 86 |
| S03 | Hot | Critical care-plastic crate housing cats in cage 11 | ~50-100 | 105 |
| S05 | Hot | Critical care-plastic crate housing cats in cage 12 | ~50-100 | 235 |
| S10 | Cold | Metal bathroom doorknob, 2^nd^ Floor | ~50 | 0 |
| S11 | Cold | Laminate table top outside warm zone, 1^st^ floor | 100 | 0 |
| S13 | Cold | Concrete floor outside warm zone, 1^st^ floor | 100 | 0 |
| S15 | Cold | Metal door latch to warm zone, 1^st^ floor | ~50 | 0 |
| S52 | Cold | Incident command laminate table top, 2^nd^ floor | 100 | 0 |
| S54 | Cold | Incident command laminate table top, 2^nd^ floor | 100 | 0 |
| S56 | Cold | Metal door handle to incident command, 2^nd^ floor | ~100 | 0 |
| S58 | Cold | Concrete floor outside warm zone, 2^nd^ floor | 100 | 0 |
| S60 | Cold | Metal elevator buttons outside incident command, 2^nd^ floor | ~50 | 0 |
| S62 | Hot | Plastic crate housing cat inside cage, Pod J | ~100 | 270 |
| S64 | Hot | In front of sick cat, surface of paper lining concrete floor inside cage, Pod J | 100 | 1880 |
| S67 | Hot | Stainless steel water bowl, Pod J | ~100 | 0 |
| S69 | Hot | Concrete floor next to bloody cat vomit, Pod J | 100 | 159 |
| S70 | Hot | Plastic dust pan near Pod J | ~100 | 0 |
| S73 | Hot | Pod G, inside floor by cat | ~100 | 1336 |
| S75 | Hot | Pod F, cardboard box with cat inside | ~100 | 689 |
| S77 | Hot | Pod F, stainless steel water bowl | ~100 | 0 |
| S79 | Hot | Pod G, paper lining concrete floor inside cage | 100 | 9516 |
| S81 | Hot | Treatment Room-laminate table top | 100 | 31 |
| S83 | Hot | Treatment Room-laminate table top | 100 | 0 |
| S85 | Hot | Critical Care-plastic crate housing cat inside cage | ~50-100 | 73 |
| S87 | Hot | Critical Care, plastic crate housing cat inside cage | ~50-100 | 67 |
| S89 | Hot | Concrete floor near exit to Warm Room, 1st floor | 100 | 4097 |
| S91 | Hot | Laminate table top near exit to warm room, 1st floor | 100 | 36 |
| S93 | Hot | Concrete floor walkway between cages in Pod C | 100 | 0 |
| S95 | Hot | Concrete floor walkway between cages in Pod C | 100 | 445 |
| S97 | Hot | Concrete floor walkway between cages in Pod D | 100 | 181 |
| S99 | Hot | Concrete floor walkway between cages in Pod D | 100 | 266 |
